# Hydrocele among Patients undergoing Surgery in the Department of Surgery in a Tertiary Care Center: A Descriptive Cross-sectional Study

**DOI:** 10.31729/jnma.8678

**Published:** 2024-07-31

**Authors:** Kishor Kumar Deo, Arun Kumar Chaudhary, Reshika Shrestha, Aashutosh Chaudhary, Bindira Adhikari, Apeksha Bista, Devesh Jha, Niliza Shakya, Suresh Maharjan, Manisha Shrestha, Ashish Shrestha, Isha Dahal, Anshu Sutihar

**Affiliations:** 1 Department of General Surgery, National Academy of Medical Sciences, Bir Hospital, Kanti Path, Kathmandu, Nepal; 2 Department of Statistics, Nepal Commerce Campus, Tribhuwan University, Minbhawan, Kathmandu, Nepal; 3 Maharajgunj Medical Campus, Institute of Medicine, Maharajgunj, Kathmandu, Nepal; 4 Kathmandu University School of Medical Sciences, Dhulikhel, Kavre, Nepal; 5 Chitwan Medical College and Teaching Hospital, Bharatpur, Chitwan, Nepal; 6 Kathmandu Medical College Public Limited, Sinamangal, Kathmandu, Nepal; 7 Venus Hospital, Midbaneshwor, Kathmandu, Nepal; 8 Department of Dental Surgery, National Academy of Medical Sciences, Bir Hospital, Kanti Path, Kathmandu, Nepal; 9 B.P Smriti Hospital, Mahrajgunj, Kathmandu, Nepal

**Keywords:** *hydrocele*, *prevalence*, *surgery*

## Abstract

**Introduction:**

Hydrocele, an accumulation of serous fluid within the remnant of the processus vaginalis, is a common cause of painless scrotal enlargement. While prevalent, few studies have been conducted to assess the extent and risk factors of hydrocele in Nepal. This study aimed to assess the prevalence and associated factors of hydrocele among patients undergoing the surgery department at a tertiary care center in Nepal.

**Methods:**

This descriptive cross-sectional study was conducted at a tertiary care center. Data were retrospectively collected from medical records over one year (2021 July to 2022 June), including all patients undergoing surgery in the general surgery department. Ethical Approval was received from the Institutional Review Committee of the same institute (Reference number: 820/2080/81) Cases of hydrocele surgery were identified, and relevant data were extracted using a structured proforma. Descriptive analyses were performed using Microsoft Excel 2016.

**Results:**

Out of 1812 surgeries, 95 (9.72%) were hydrocele surgeries. Of these, 94 (98.95%) were non-communicating hydroceles, 79 (83.16%) were unilateral, and 90 (94.74%) showed positive transillumination tests. The mean age of patients was 50.84 ± 17.02 years, with the highest number of cases in the 46-55 age group (20%). Postoperative complications occurred in 19 (20%) patients, with seroma and surgical site infection being the most common (31.58% each).

**Conclusions:**

Hydrocele surgeries comprised a significant portion (5.24%) of surgical cases at the tertiary care center, with the majority being non-communicating and unilateral types.

## INTRODUCTION

Hydrocele is an accumulation of serous fluid within the remnant of the processus vaginalis, which can be congenital or acquired.^1^ It is the most prevalent cause of painless scrotal enlargement and affects around 1% of men over 40 years old and 4.7% of newborns.^2^ Hydroceles can either be congenital or acquired. The majority of acquired hydroceles are idiopathic, while they can also result from neoplasms, intrascrotal infections, regional or systemic illnesses, inguinal or scrotal surgery, or infections.^3^ Chronic hydrocele is quite common, particularly in filariasis-endemic locations where *Wuchereria bancrofti* is the causal agent.^4^ Surgery and aspiration are both used as forms of treatment, either with or without sclerotherapy.^5^

Despite the prevalent incidence and clinical relevance of hydrocele, relatively few studies exist in Nepal. Hydrocele prevalence increases with age, particularly in Asian and African regions, reaching up to 50% in those over 45 years causing physical disability, social stigma and reduced economic contribution which reflects the importance of hydrocele repair which is available free of cost in all government hospitals of Nepal.^6^

This study aimed to assess the prevalence of hydrocele among patients visiting the surgery department at the tertiary care center of Nepal.

## METHODS

This descriptive cross-sectional study was conducted among patients undergoing surgery in the Department of General Surgery of National Academy of Health Sciences, Bir Hospital. The process of data collection was started after obtaining ethical approval from the Institutional Review Committee of the National Academy of Health Sciences (Reference number: 820/2080/81). The data was collected retrospectively from the medical records of approximately one year from 2021 July to 2022 June. All the patients undergoing surgery in the general surgery department were included in this study whereas those entries with missing data were excluded from the study. Total sampling was done.

Cases of hydrocele surgery were identified, and relevant data were collected using a structured proforma. The variables collected included age, method of treatment, type of hydrocele, onset, swelling, fluctuation, transillumination test results, pain, and postoperative complications.

The data were entered and analyzed using Microsoft Excel 2016. Descriptive analyses were done in which percentages were calculated for binary data, and mean and standard deviation were calculated for continuous data. Point estimates at 95% Confidence Interval (CI) were calculated.

## RESULTS

A total of 1812 surgeries were performed during the data collection duration where 977 (53.91%) were male and 835 (45.92%) were female. Out of the total surgery, 95 (9.72%) (7.86-11.58, 95% CI) hydrocele surgery were performed. Among total cases, 94 (98.95%) were communicating and one (1.05%) was non-communicating hydrocele. There were 79 (83.16%) unilateral cases where 41 (51.90%) were right sided and remaining 38 (48.10%) were left sided hydrocele. A total of 90 (94.74%) showed a positive transillumination test (Table 1).

**Table 1:** Details of hydrocele (n = 95).

Variables	n (%)
**Type of hydrocele**	
Non-communicating	94 (98.95)
Communicating	1 (1.05)
**Laterality**	
Unilateral	79 (83.16)
Right	41 (51.90)
Left	38 (48.10)
Bilateral	16 (16.84)
**Transillumination test**	
Positive	90 (94.74)
Negative	5 (5.26)

The median of the patients undergoing hydrocele surgery was 50 years (Q3-Q1, 64.5-38), ranging from 15 to 86. A total of 67 (70.50%) patients operated from inside the valley (Table 2). There were 18 (18.95%) patients in the 36-45 age group and 1 (1.05%) from 8695 age group. A total of 67 (70.50%) patients operated from inside the valley.

**Table 2:** Demographic details of patients undergoing hydrocele surgery (n = 95).

Demographic parameters	n (%)
**Age group**	
15-25	7 (7.39)
26-35	12 (12.63)
36-45	18 (18.95)
46-55	19 (20)
56-65	16 (16.84)
66-75	16 (16.84)
76-85	6 (6.32)
86-95	1 (1.05)
**Residence**	
Inside valley	67 (70.50%)
Outside valley	28 (29.50%)

Postoperative complications were present in 19 (20%) of the patients undergoing hydrocele surgery. Seroma and surgical site infection was seen in 6 (31.38%) each (Table 3).

**Table 3:** Postoperative complications of hydrocele (n = 19).

Postoperative complications	n (%)
Seroma	6 (31.58)
Surgical site infection	6 (31.58)
Hematoma	3 (15.79)
Chronic pain	2 (10.53)
Wound dehiscence	2 (10.53)

## DISCUSSION

Hydrocele repair is one of the most common general surgical procedures performed, however, its prevalence in developing countries like Nepal is underreported in the literature.^1^ In our study among the total surgeries performed in our institute in a year, there were a total of 9.72% hydrocele. Hydrocele is the accumulation of serous fluid between visceral and parietal layers of the tunica vaginalis of testes which is further divided into primary and secondary based upon pathophysiology.^7^ Primary hydrocele might be congenital or neonatal, communicating and non-communicating or closed type while secondary hydroceles develops as a complication of pre-existing disease but most commonly, the cause is idiopathic.^3,7^ Hydrocele is the most common cause of painless scrotal swelling.^2^ Cystic intra-scrotal conditions, such as hydroceles and spermatoceles, are commonly encountered in general urological practice.^5^ Surgery is the recommended treatment for hydrocele and is available free of cost in all government hospitals in Nepal.^6^

A communicating hydrocele resembles a hernia, but the sac connecting the abdomen to the scrotum or labia majora contains only fluid, not abdominal contents. In contrast, a non-communicating hydrocele is an isolated collection of scrotal fluid with no connection to the abdomen.^4^ Noncommunicating hydroceles are among the most prevalent forms of hydrocele worldwide, impacting over 30 million men and boys.^8^ Similar to this, our study revealed that noncommunicating hydroceles are more prevalent than communicating ones. A retrospective study done by Li P et al revealed a total 138 cases of non-communicating hydrocele during a 5 month duration in children between 11 to 72 months which is more than in our study.^9^

Hydrocele in children is commonly caused by failure of obliteration of processus vaginalis either partially or completely whereas in adults, it is usually acquired due to the disruption of lymphatic system by torsion of the testicle, lymphoma, or the death of parasitic filarial worm.^1,8,10^ Surgeries like laparoscopic varicocelectomy can interfere in testicular lymphatic drainage leading to the hydrocele as postoperative complication.^10^ Another cause of hydrocele is the imbalance between drainage and fluid input into the lymphatic tissue surrounding the scrotum.^11^ The age distribution of hydroceles and spermatoceles in the current study clearly indicates that these conditions are more prevalent in older men. However, hydroceles in childhood are common with a completely different pathogenesis. Similarly, in our study among the 95 hydrocele cases studied, the age distribution skewed more towards adults than children.^5^ Hydroceles are often unilateral and right sided with 7-10% prevalence of bilateral hydrocele which is similar to our study.^12^

Hydrocele can be diagnosed based on clinical evaluation, including patient history and physical examination. Majority of the hydrocele cases are asymptomatic. Larger hydroceles may cause chronic pain in the scrotum or lower back and can injure scrotal contents, including the testicle.^2^ Examination should be done in both supine and upright position since hydrocele gets reduced depending on the position.^13^ Transillumination of the hemiscrotum confirms the diagnosis. If light passes through the scrotum during transillumination, it is widely believed that the mass is cystic. Conversely, if the light is blocked, the mass is considered solid although some hydroceles do not transilluminate due to thickening of the tunica vaginalis which prevents accurate hydrocele identification through transillumination.^10,12^ Likewise, in our study, the transillumination test was positive in 90 patients and the rest were negative. The most causes for negative transillumination tests are infected hydrocele and thickened and calcified sacs of hydrocele. In our cases transillumination test was negative probably due to long standing hydrocele where the sac has become thickened and calcified. Confirmatory USG is done in all cases.

Duplex Doppler sonography is the recommended diagnostic tool which is effective for detecting hydrocele in patients with intrascrotal calcifications or non-palpable hydroceles.^10^ Ultrasonography offers a simple, noninvasive imaging tool that can eliminate the need for fluid aspiration which is anyway considered a potential hazard of tumor spillage for patients without a clear cause for hydrocele in their history and prevents misdiagnosis particularly when hydrocele is accompanied by tumours.^10,14^ A computed tomography (CT) scan with and without contrast is an appropriate imaging study for patients with complex, undiagnosed, or difficult-to-diagnose hydrocele on ultrasound whereas 1-2 ml of serous fluid may be present in the potential cavity of the tunica vaginalis of a normal scrotum which should not be mistaken for a hydrocele.^10,13^ The volume of tunica vaginalis fluid required to define a hydrocele remains unclear. Even in obvious cases, the amount of fluid can vary significantly, ranging from a small, soft swelling to a large, firm scrotal lump.A study done by Leung et al. revealed minimal amount of fluid in one hemiscrotum in 86% of their healthy group of volunteers.^2^

Hydrocele is treated basically by draining the excessive fluid and preventing reaccumulation either by aspiration with sterile syringe who cannot tolerate surgery or by sclerotherapy or hydrocelectomy to eliminate space between parietal and visceral layer of tunica vaginalis.^14,15^ Congenital hydroceles typically resolve spontaneously by the end of the first year of life but spontaneous resolution of acquired hydrocele is rare.^1,15^ Herniotomy is performed for congenital hydrocele if not resolved spontaneously with a high success rate. To minimize operative risk in congenital hydrocele, caution should be exercised before rushing neonates and infants to surgery.^1^ However, we could not reflect the data on congenital hydrocele as in our center, surgical treatment is only performed for those aged 15 years and older. The prognosis for congenital hydrocele is excellent, while the outcome for adult-onset hydrocele depends upon underlying cause.^15^

Following aspiration, risk of hematocele and infection as well as reaccumulation within a week is high.^15^ Similarly, other postoperative complications of hydrocele surgery include hematoma, seroma, infection, pyocele and wound dehiscence.^15,16^ A retrospective study done by Maki-Lohiluoma L et al identified 866 hydrocele surgeries in 9 years duration with post surgical complications in 139 patients (16.1%). Similarly, in our study, postoperative complications were present in 19 (20%) patients undergoing hydrocele surgery which is near to the study findings done by Maki-Lohiluoma L.^16^ When treating hydroceles, physicians should consider factors such as success rate, morbidity, costs to the patient, the healthcare system, the community, and patient satisfaction.

This study fails to include all the patients with hydrocele who have been diagnosed in the outpatient department and did not undergo surgery, hence true prevalence of hydrocele is underestimated as a result of selection bias. However, using the entire population database without selecting for different study variables highlights the importance of this study.

## CONCLUSIONS

The study found that hydrocele is a relatively common condition requiring surgical intervention, accounting for 5.24% of surgeries in the general surgery department. The majority of hydroceles were of the communicating type and unilateral. Postoperative complications, including seroma and surgical site infections, were notable, occurring in 20% of cases.
